# CULTURAL FACTORS PREDICT POSITIVE CAREGIVING APPRAISAL AMONG DIVERSE DEMENTIA FAMILY CAREGIVERS

**DOI:** 10.1093/geroni/igad104.2522

**Published:** 2023-12-21

**Authors:** Michael McCarthy, Mark Remiker, Y Evie Garcia, Heather Williamson, Julie Baldwin

**Affiliations:** Northern Arizona University, Flagstaff, Arizona, United States; Northern Arizona University, Flagstaff, Arizona, United States; Northern Arizona University, Flagstaff, Arizona, United States; Northern Arizona University, Flagstaff, Arizona, United States; Northern Arizona University, Flagstaff, Arizona, United States

## Abstract

**Background and Objectives:**

Positive caregiving appraisal is strongly linked to health among caregivers of persons with Alzheimer’s Disease and Related Dementias (ADRD). Few studies have identified predictors of positive caregiving appraisal, although research suggests that cultural factors may play a role. This study hypothesized that self-identified race and ethnicity, as well as more nuanced cultural factors such as language spoken at home, culture-based values surrounding caregiving, and perceptions of provider cultural competence would predict positive caregiving appraisal.

**Research Design and Methods:**

A cross-sectional survey was administered to 156 diverse, community dwelling ADRD caregivers. Data were analyzed according to a modified Sociocultural Model of Coping using sequential regression.

**Results:**

In the final model, self-identified race and ethnicity did not predict positive appraisal. However, culture-based values around caregiving and provider cultural competence contributed significant variance to positive appraisal, after controlling for other factors in the model. Caregiving burden and perceptions that help was available were also linked to positive appraisal. Discussion and Implications: This study is among the first to identify cultural factors that contribute to positive appraisal in a diverse sample of ADRD caregivers, beyond race and ethnicity. Findings reinforce the need to assess and incorporate culture-based values in interventions for ADRD caregivers, as well as the importance of provider cultural competence when working with diverse ADRD family caregivers.

